# Manifold implications of obesity in ischemic heart disease among Japanese patients according to covariance structure analysis: Low reactivity of B-type natriuretic peptide as an intervening risk factor

**DOI:** 10.1371/journal.pone.0177327

**Published:** 2017-05-08

**Authors:** Joshi Tsutsumi, Kosuke Minai, Makoto Kawai, Kazuo Ogawa, Yasunori Inoue, Satoshi Morimoto, Toshikazu Tanaka, Tomohisa Nagoshi, Takayuki Ogawa, Michihiro Yoshimura

**Affiliations:** Division of Cardiology, Department of Internal Medicine, The Jikei University School of Medicine, Tokyo, Japan; Scuola Superiore Sant'Anna, ITALY

## Abstract

**Background/Objectives:**

Obesity is believed to be one of the major risk factors for cardiovascular disease in Western countries. However, the effects of obesity should be continuously examined in the Japanese population because the average bodily habitus differs among countries. In this study, we collectively examined the significance of obesity and obesity-triggered risk factors including the low reactivity of B-type natriuretic peptide (BNP), for ischemic heart disease (IHD) in Japanese patients.

**Methods and results:**

The study patients consisted of 1252 subjects (IHD: n = 970; non-IHD: n = 282). Multiple logistic regression analysis revealed that dyslipidemia, hypertension, diabetes, and the low reactivity of BNP were significant risk factors for IHD, but body mass index (BMI) was not. A theoretical path model was proposed by positioning BMI at the top of the hierarchical model. Exploratory factor analysis revealed that BMI did not play a causative role in IHD (P = NS). BMI was causatively linked to other risk factors (P<0.001 for hypertension; P<0.001 for dyslipidemia; P<0.001 for HbA1c; P<0.001 for LogBNP), and these factors played a causative role in IHD (P<0.001 for hypertension; P<0.001 for dyslipidemia; P<0.001 for HbA1c; P<0.001 for LogBNP). The intrinsic power of the low reactivity of BNP induced by high BMI on the promotion of IHD was fairly potent.

**Conclusion:**

This study demonstrated that obesity per se is not a strong risk factor for IHD in Japanese patients. However, several important risk factors triggered by obesity exhibited a causative role for IHD. The low reactivity of BNP is a substantial risk factor for IHD.

## Introduction

Metabolic syndrome is a cluster of metabolic risk factors and is a crucially important pathological condition for the progression of ischemic heart disease (IHD) and other cardiovascular diseases [1–3]. Obesity may be the most influential risk factor for metabolic syndrome. A central feature of obesity or metabolic syndrome is insulin resistance, which results in hyperglycemia and hyperinsulinemia and eventually leads to the development of diabetes [4]. Continuous low-grade inflammation is another feature of metabolic syndrome [5], and it results in complex metabolic abnormalities that contribute to the pathogenesis of hypertension, dyslipidemia, and diabetes. Therefore, obesity and metabolic syndrome are associated with atherosclerosis [6, 7].

Obesity plays a primary role in metabolic syndrome [8, 9], and it frequently causes IHD. Although the direct effect of obesity on the progression of IHD is not as clear in the Japanese population, obesity-triggered risk factors, such as diabetes, hypertension, and dyslipidemia, are clearly documented detrimental factors [10]. If the degree of obesity is not severe, adipose tissue is likely a valuable energy source. Low body mass index (BMI) or low cholesterol may also be detrimental and associated with higher mortality in patients with heart failure [11]. Therefore, mild obesity, which is common in Japan, may be preferable rather than detrimental to health, although the optimal weight or its upper threshold is not clear in individual patients. There is a need to elucidate the complex relationship between obesity and obesity-related disorders as risk factors for IHD in the Japanese population.

B-type natriuretic peptide (BNP) is a hormone secreted from the ventricles, and plasma levels of BNP reflect the degree of heart failure [12]. Numerous factors increase the secretion of BNP, such as ventricular mechanical stretch, many neurohumoral factors, and inflammation [13, 14]. In contrast, few factors reduce plasma BNP levels. Obesity may be an important factor for reducing plasma BNP levels [15], and some genetic contributors may exist in the BNP gene sequence [16–18]. A factor that reduces BNP production or augments BNP clearance may not increase plasma BNP levels despite the presence of cardiac hemodynamic overload. The low reactivity of BNP, which can be called a natriuretic handicap [19], would augment the severity of heart failure and other disorders via disruption of the hormonal balance of the renin-angiotensin-aldosterone and sympathetic nervous systems, among other effects [20]. The possible interaction between obesity and the plasma BNP levels is important to discuss in the clinical setting. We recently reported a significant association of the low reactivity between BNP and IHD [21], although the causality remains unclarified.

Obesity would be the leading cause of IHD, but how obesity per se or obesity-triggered risk factors produce IHD is not clear. The most difficult aspect of this type of study is designing the study for the simultaneous comparison of multiple risk factors for IHD by constructing a hierarchical model. To the best of our knowledge, there are no reports using this type of research plan and complex statistical analysis. Simultaneous analysis would provide a deeper understanding of the characteristics of obesity per se and obesity-triggered disorders as the risk factors for IHD.

Covariance structure analysis plays an important role in understanding how the relationships between observed variables may be generated using hypothesized latent variables in many areas. Covariance structure analysis is useful for exploratory and explanatory factor analyses. However, the input factors should be carefully selected when planning covariance structure analyses, and the path model based on covariance structure analysis should be proposed on the basis of scientific knowledge, abundant experience, consistent concepts and the clear direction of the study. We recently proposed path models to explain complex phenomena, including plasma BNP levels [22].

In the present study, we primarily compared IHD with non-IHD using possible valuables in a conventional multiple logistic regression analysis. We secondarily developed a complex statistical analysis using covariance structure analysis to examine the power of obesity per se and obesity-triggered disorders on IHD and non-IHD in a single-equation model.

## Materials and methods

### Study patients

Study patients consisted of 1252 subjects who were consecutively admitted to our institutions from 2012 to 2015 because of some type of heart disorder. Acute coronary syndrome was excluded from this study because plasma BNP levels are greatly altered after the onset of acute myocardial infarction, as shown in our previous report [23]. Therefore, we recruited stable patients with IHD in this study. The Ethics Committee of The Jikei University School of Medicine approved the study protocol (24–355[7121]), and we complied with the routine ethical regulations of our institution. Informed consent was obtained from each patient, and all clinical investigations were conducted in accordance with the principles expressed in the Declaration of Helsinki. We also posted a notice about the study design and contact information at a public location in our institution.

### Definition of diseases

IHD was diagnosed using symptoms, electrocardiography, blood sampling and morphology of the coronary arteries. Patients with IHD included patients with a clinically stable phase of IHD. Organic stenosis was defined as 75% or greater in the coronary arteries on coronary angiography. Patients with coronary spastic angina were included in the IHD group of this study if disease activity was stable; if coronary spasm was previously diagnosed; or if the provocation test was planned during this hospitalization. Valvular diseases included heart failure caused by moderate valvular disease and patients who were scheduled for surgery. Arrhythmia included the need for catheter ablation, an implantable cardioverter-defibrillator, cardiac resynchronization therapy and patients with a pacemaker or syncope. Cardiomyopathy was defined in patients who were diagnosed before admission and were undergoing treatment or who were diagnosed after admission. Conventional risk factors were defined using well-known criteria. Briefly, hypertension was defined as systolic blood pressure ≥140 mmHg or diastolic blood pressure ≥90 mmHg or use of antihypertensive agents. Dyslipidemia was diagnosed by the presence of more than one of following lipid disorders at the first fasting blood sampling or use of lipid-lowering agents. We defined high LDL cholesterol as LDL cholesterol levels ≥140 mg/dL, hypertriglycemia as triglyceride levels ≥150 mg/dL, and low HDL cholesterol as HDL cholesterol levels <40 mg/dL. Diabetes was defined as the presence of any of the following factors: fasting plasma glucose levels ≥126 mg/dL, casual plasma glucose levels ≥200 mg/dL, HbA1c (NGSP) ≥6.5% and medical history of diabetes.

### Blood sampling and measurement of biochemical examination

We collected blood samples and hemodynamic data during cardiac catheterization. Routine biochemical analyses, such as electrolytes, renal function, liver function and lipid profiles, were performed in a central laboratory in our hospital during the study. The measurement of plasma BNP levels was also simultaneously performed as described in previous reports [20–22,24]. In brief, whole blood (5 mL) was collected in tubes containing potassium ethylenediaminetetraacetic acid (1 mg/mL blood). The plasma BNP level was then measured as the immune-reactive circulating levels of BNP with a rapid enzyme-linked immunosorbent assay (non-extracted) kit using an antibody to human BNP (Shionogi Co. Ltd., Tokyo, Japan).

### Statistical analysis

Continuous variables are expressed as the means ± standard deviation (SD) or medians. Categorical variables are expressed as percentages. Comparisons between groups were performed using Pearson’s chi-square test for categorical variables and the Mann-Whitney U test or Student’s t-test for continuous variables. The Kolmogorov-Smirnov test was used to determine whether the BNP values were normally distributed. Subsequently, the BNP data were log- transformed (LogBNP) to achieve a normal distribution for the analysis. A multivariate logistic regression analysis was also used to determine whether IHD was present. Statistical analyses were performed using SPSS Statistics version 23.0 (SPSS Inc., Chicago, IL, USA). A path model based on covariance structure analysis was proposed to investigate the relationship among clinical factors in this study population and survey probable causal effects on IHD. Path analysis was performed using IBM SPSS AMOS version 23 (Amos Development Corporation, Meadville, PA, USA). Dyslipidemia (0 for absence and 1 for presence), hypertension (0 for absence and 1 for presence), diabetes (0 for absence and 1 for presence), and IHD (0 for non-IHD and 1 for IHD) were included as variables in the model. The obtained structural equation models were tested and confirmed at the significance level for P values<0.05. The causality model defines hierarchical regression models between clinical factors and IHD. Paths between variables are drawn from independent to dependent variables with directional arrows for every regression model (arrowhead on one end only). A two-way arrow between two variables indicates a correlation between those two variables. The total variance in a dependent variable for every regression is theorized to be caused either by independent variables of the model or extraneous variables (e). Each path has a coefficient showing the standardized coefficient of the regressing independent variable on the dependent variable of the relevant path. The indirect effect was determined by multiplying the path coefficients of intervening variables. All statistical analyses including the obtained structural equation models were tested and confirmed at a P value significance level of <0.05.

## Results

### Study patients’ characteristics

Table 1 shows the patients’ characteristics in this study. A total of 970 of the 1252 patients were diagnosed as having IHD, and 282 patients were non-IHD. Characteristic findings, included higher age and a higher male-to-female ratio in the IHD group than in the non-IHD group. The plasma BNP level was significantly lower and the left ventricular ejection fraction (LVEF) was significantly higher in the IHD group than in the non-IHD group. Additionally, the HbA1c level was significantly higher in the IHD group than in the non-IHD group.

**Table 1:** Clinical baseline characteristics of the study subjects.

	IHD group (n = 970)	non-IHD group (n = 282)	P value
**Age (yrs)**	65.9±10.5	62.7±13.1	P<0.01
**Men (%)**	86.6	69.9	P<0.01
**BMI (kg/m**^**2**^**)**	24.6±3.8	23.4±4.5	P<0.01
**DM (%)**	41.4	21.3	P<0.01
**DLP (%)**	81.9	45.0	P<0.01
**HT (%)**	75.7	61.0	P<0.01
**Current+Past smoker (%)**	71.4	48.9	P<0.01
**Current smoker (%)**	21.1	20.6	NS
**s-Cr (mg/dL)**	0.88±0.53	0.85±0.22	NS
**HbA1c (NGSP) (%)**	6.3±1.0	5.9±0.9	P<0.01
**BNP (ng/L)**	76.4±126.7	170.4±220.9	P<0.01
**BNP (ng/L) Median (interquartile range)**	32.3(14.3–83.8)	95.3(29.7–208.7)	P<0.01
**LogBNP (ng/L)**	1.56±0.52	1.91±0.59	P<0.01
**LVEF (%)**	58.6±10.5	51.3±15.1	P<0.01
**Heart rate (beats/min) (at Post-LVG)**	69.7±11.4	75.2±16.1	P<0.01
**LVEDP (mmHg) (at Pre-LVG)**	15.9±8.9	17.6±8.0	P<0.01
**LVEDVI (mL/m**^**2**^**)**	63.6±18.1	84.1±34.8	P<0.01
**LVESVI (mL/m**^**2**^**)**	27.3±14.8	43.6±28.2	P<0.01
**OMI (%)**	36.7	0.0	P<0.01
**Prior PCI (%)**	49.5	0.0	P<0.01
**Prior CABG (%)**	10.1	0.0	P<0.01
**Prior valve repair (%)**	0.7	1.8	NS
**Valvular heart disease (%)**	5.5	36.2	P<0.01
**Congenital heart disease (%)**	0.4	4.3	P<0.01
**Cardiomyopathy (%)**	2.1	38.3	P<0.01
**Atrial fibrillation (%)**	3.7	20.2	P<0.01
**Calcium-channel blockers (%)**	64.0	32.6	P<0.01
**ACE-inhibitors (%)**	23.5	14.5	P<0.01
**ARBs (%)**	41.9	28.4	P<0.01
**Nitrates (%)**	21.6	4.6	P<0.01
**Nicorandil (%)**	13.3	3.9	P<0.01
**Beta-blockers (%)**	44.6	28.4	P<0.01
**Statins (%)**	72.7	22.0	P<0.01
**Diuretics (%)**	16.4	30.9	P<0.01
**OHA (%)**	29.2	11.0	P<0.01
**Insulin (%)**	11.0	4.6	P<0.01

BMI, Body mass index, DM, Diabetes mellitus; DLP, Dyslipidemia; HT, Hypertension; s-Cr, Serum-creatinine; BNP, B-type natriuretic peptide; LVG, Left Ventriculography; LVEF, Left ventricular ejection fraction; LVEDP, Left ventricular end diastolic pressure; LVEDVI, Left ventricular end-diastolic volume index; LVESVI, Left ventricular end-systolic volume index; OMI, Old myocardial infarction; PCI, Percutaneous coronary intervention; CABG, Coronary artery bypass graft; ACE, Angiotensin converting enzyme; ARBs, Angiotensin receptor blockers; OHA, Oral hypoglycemic agents.

### Multiple logistic regression analysis for determinations of IHD compared with non-IHD

Multivariable logistic regression analysis was performed to examine the effect of BMI on IHD compared with non-IHD. The analysis identified the factors of dyslipidemia, hypertension, and diabetes as significant factors for IHD (P<0.01) (Table 2). BMI exhibited a similar tendency, but the effect was not statistically significant (P = NS (0.087)). The next multiple logistic regression analysis included LogBNP as one of the explanatory factors because the low reactivity of BNP was a possible cardiovascular risk. Table 3 shows that the contributions of dyslipidemia, hypertension, and diabetes were highly significant for a determination of IHD (P<0.01) and that the low reactivity of BNP was a significant risk for IHD (P<0.01). BMI was not significant (P = NS (0.825)). This result suggests that the low reactivity of BNP may play a role in obesity.

**Table 2:** Multiple logistic regression analysis for IHD.

Explanatory variable	Regression coefficient	P value	Odds ratio	Odds 95% CI
**BMI**	0.035	NS (P = 0.087)	1.035	0.995–1.077
**DLP**	1.538	<0.01	4.654	3.463–6.256
**HT**	0.46	<0.01	1.584	1.163–2.157
**HbA1c (NGSP)**	0.366	<0.01	1.442	1.204–1.728

BMI, Body mass index; DLP, Dyslipidemia; HT, Hypertension.

**Table 3:** Multiple logistic regression analysis for IHD with involvement of BNP.

Explanatory variable	Regression coefficient	P value	Odds ratio	Odds 95% CI
**BMI**	0.005	NS (P = 0.825)	1.005	0.965–1.046
**DLP**	1.406	<0.01	4.079	3.009–5.528
**HT**	0.536	<0.01	1.71	1.243–2.352
**HbA1c (NGSP)**	0.396	<0.01	1.486	1.237–1.786
**Log BNP**	-1.011	<0.01	0.364	0.275–0.481

BMI, Body mass index; DLP, Dyslipidemia; HT, Hypertension; BNP, B-type natriuretic peptide

### Result of path model A

Theoretical path model A was proposed with other risk factors by positioning BMI at the top. Obesity-triggered risk factors, such as dyslipidemia, hypertension, diabetes, and low-reactive BNP, were placed below BMI. Table 4 shows the precise results of path model A. The exploratory factor analysis revealed that BMI did not play a causative role in IHD (standardized regression coefficient, β: -0.003, P = NS). However, BMI was causatively linked to other risk factors (β: 0.165, P<0.001 for hypertension; β: 0.152, P<0.001 for dyslipidemia; β: 0.190, P<0.001 for HbA1c; β: -0.217, P<0.001 for LogBNP), and these factors played a causative role in IHD (β: 0.095, P<0.001 for hypertension; β: 0.279, P<0.001 for dyslipidemia; β: 0.112, P<0.001 for HbA1c; β: -0.207, P<0.001 for LogBNP).

**Table 4:** Results of path model A (Fig 1).

		Clinical factor	Direct effect	Indirect effect	Total effect	P value
**IHD**	←	**BMI**	-0.003	0.124	0.121	P = 0.898
**DLP**	←	**BMI**	0.152	0.000	0.152	P<0.001
**LogBNP**	←	**BMI**	-0.217	0.000	-0.217	P<0.001
**HT**	←	**BMI**	0.165	0.000	0.165	P<0.001
**HbA1c**	←	**BMI**	0.19	0.000	0.190	P<0.001
**IHD**	←	**DLP**	0.279	0.000	0.279	P<0.001
**IHD**	←	**LogBNP**	-0.207	0.000	-0.207	P<0.001
**IHD**	←	**HT**	0.095	0.000	0.095	P<0.001
**IHD**	←	**HbA1c (NGSP)**	0.112	0.000	0.112	P<0.001

IHD, Ischemic heart disease; BMI, Body mass index; DLP, Dyslipidemia; BNP, B-type natriuretic peptide; HT, Hypertension.

**Fig 1:**
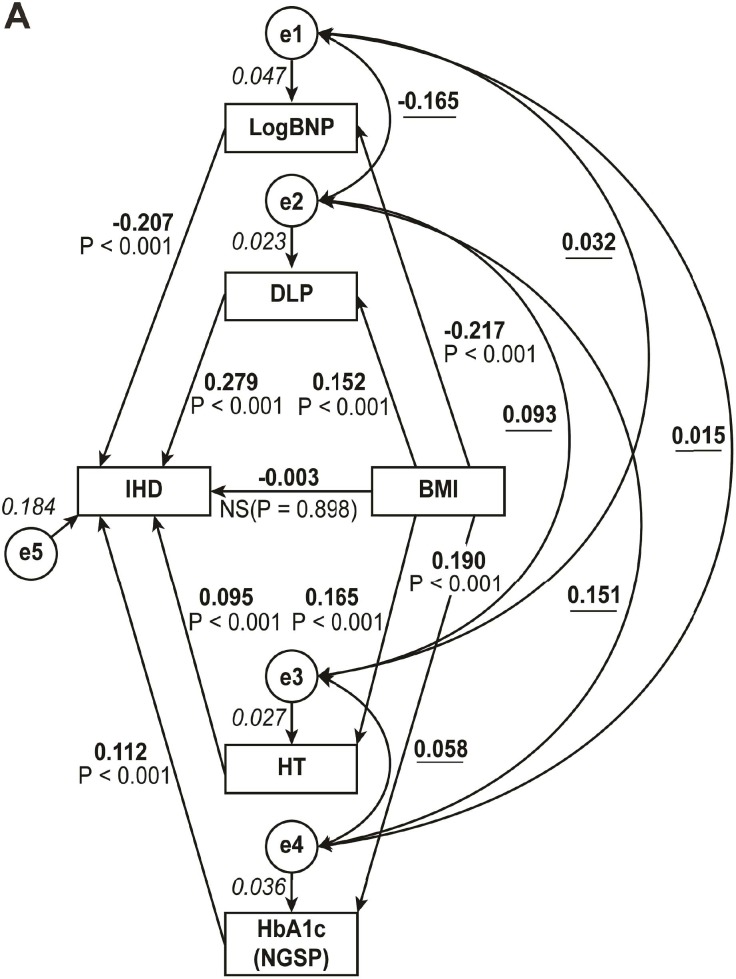
Path model A: Explanatory drawing of possible cascade from BMI to IHD directly and via the low reactivity of BNP, dyslipidemia, hypertension, and HbA1c. This path has a coefficient showing the standardized coefficient of regressing independent variables on the dependent variable of the relevant path. These variables indicate standardized regression coefficients (direct effect) [simple capitals], squared multiple correlations [narrow italic capitals] and correlations among exogenous variables [capitals inside round brackets]. A two-way arrow between two variables indicates a correlation between those two variables. The total variance in a dependent variable for every regression is theorized to be caused by either independent variables of the model or extraneous variables (e). BMI: body mass index; BNP: B-type natriuretic peptide; e: extraneous variable.

### Concept of proposed path model B1 and its result

**Fig 2:**
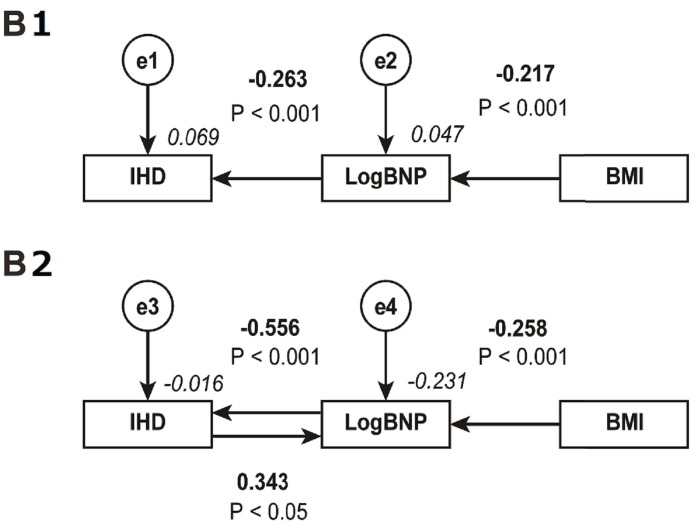
Path models B1 and B2: explanatory drawing of Ppossible cascade from BMI to BNP and further to IHD. This path has a coefficient showing the standardized coefficient of regressing independent variables on the dependent variable of the relevant path. These variables indicate standardized regression coefficients (direct effect) [simple capitals], squared multiple correlations [narrow italic capitals] and correlations among exogenous variables [capitals inside round brackets]. BMI; body mass index; BNP: B-type natriuretic peptide; IHD: ischemic heart disease; e: extraneous variable. B1. Path model B1: a simple path model for the connection between BNP as a cause and IHD as an effect. B2. Path model B2: directional paths between BNP and IHD to distinguish between cause and effect.

The covariance structure analysis revealed a potential power of the low reactivity of BNP in the promotion of IHD. Fig 2 (upper) shows the next path model, B1, proposed for a more precise evaluation. This model was just a portion of path model A. We simplified the paths from BMI to LogBNP and further to IHD. Table 5 shows the results of statistical analyses. Path model B1 revealed a significant association from BMI to LogBNP (β: -0.217, P<0.001). The model also revealed an inverse cascade from LogBNP to IHD (β: -0.263, P<0.001). This simple path model suggested that the low reactivity of BNP was an important factor that intervened between BMI and IHD.

**Table 5:** Results of path model B1 (Fig 2).

		Clinical factor	Standardized regression coefficient	P value
**LogBNP**	←	**BMI**	-0.217	P<0.001
**IHD**	←	**LogBNP**	-0.263	P<0.001

BNP, B-type natriuretic peptide; BMI, Body mass index; IHD, Ischemic heart disease.

### Concept of proposed path model B2 and its result

The contribution of the low reactivity of BNP to IHD was statistically significant but still seemingly weak because the standardized regression coefficient (β) was only -0.263. Fig 2 (lower) shows the next planned path model, B2. The purpose of this model was to examine the cause and effect between the low reactivity of BNP and IHD progression. Ischemia in the heart increases BNP production, which may be a positive feedback control. Table 6 shows the results of statistical analyses. The model revealed a significant association from LogBNP to IHD with an inverse correlation (β: -0.556, P<0.001) and a cascade from IHD to LogBNP with a positive correlation (β: 0.343, P<0.05). This result revealed why only a weak cascade was observed from LogBNP to IHD in path model B1 (β: -0.263). The contribution of LogBNP to IHD should have balanced out, and the intrinsic power of the effect of the low reactivity of BNP on the promotion of IHD was fairly strong (β: -0.556).

**Table 6:** Results of path model B2 (Fig 2).

		Clinical factor	Standardized regression coefficient	P value
**LogBNP**	←	**BMI**	-0.258	P<0.001
**IHD**	←	**LogBNP**	-0.556	P<0.001
**LogBNP**	←	**IHD**	0.343	P<0.05

BNP, B-type natriuretic peptide; BMI, Body mass index; IHD, Ischemic heart disease.

### Additional path analysis by gender difference

A theoretical path model (similar to path model A) was proposed for each gender. The results of the gender-segregated analysis appeared similar to the analysis of the total study population. In essence, among males (840 in the IHD group and 197 in the non-IHD group), the exploratory factor analysis revealed that BMI did not play a causative role in IHD (β: -0.036, P = NS), that BMI was causatively linked to LogBNP (β: -0.183, P<0.001), and that LogBNP played a causative role in IHD (β: -0.201, P<0.001). Among females (130 in the IHD group and 85 in the non-IHD group), the exploratory factor analysis revealed that BMI did not play a causative role in IHD (β: 0.003, P = NS), that BMI was causatively linked to LogBNP (β: -0.214, P = 0.001), and that LogBNP played a causative role in IHD (β: -0.140, P<0.05 for LogBNP).

## Discussion

### Multiple logistic regression analysis using conventional risk factors and inclusion of BNP for determinations of obesity as a cause of IHD

The current study demonstrated that dyslipidemia, hypertension, and diabetes were strongly correlated with IHD using multiple logistic regression analysis and that obesity per se was not clearly related to IHD (P = NS (0.087)). Thus, the contribution of obesity to IHD is still unclear. The reason is unknown; however, we speculate a possible contribution of low responsibility of BNP.

It is widely accepted that plasma BNP increases as a consequence of heart failure and is a useful marker of the degree of heart failure [12, 13]. However, some patients exhibit plasma BNP levels that are inadequate to compensate for the degree of heart failure. This hormonal imbalance may cause an advancement of atherosclerosis, IHD, and heart failure [19–21]. Furthermore, we recently reported that the plasma BNP levels were relatively lower in IHD patients than in non-IHD patients [21], although the causality was unclear. To investigate the possibility of a BNP contribution, we used BNP as one factor in the current multiple logistic regression analyses and demonstrated that the low reactivity of BNP was a significant determinant of IHD. Notably, the effect of BMI on IHD almost completely disappeared (P = NS (0.825)) when BNP was included in the equation model.

### The necessity of covariance structure analysis and the proposed theoretical path model

We consider the current multiple logistic regression analysis to have two limitations. One limitation is that the explanatory valuables confounded each other as little as possible. For example, an inverse correlation between BMI and LogBNP was identified using a single regression analysis as a reference example (N = 1252, R = -0.217, P<0.01). The other limitation is that multiple logistic regression analysis is not a hierarchical structural model, and obesity should be positioned ahead of other risk factors from the perspective of metabolic syndrome.

The precise path model A was successfully tested in this study. We elicited two findings using this highly statistical model. First, this model clearly demonstrated that obesity per se was not a significant risk factor for IHD in this population, but the obesity-triggered risk factors were substantially important for IHD. Second, the low reactivity of BNP was a significant risk factor for IHD. The covariance structure models reinforced the results obtained in the multiple regression analysis with great accuracy.

### Strong but concealed power of the low reactivity of BNP in the progression of IHD

The relation between the low reactivity of BNP and progression of IHD should be examined in more detail. Path model B1 was a portion of path model A. This model demonstrated an inverse link from LogBNP to IHD with high statistical significance (P<0.001). However, we considered the contribution, (β: -0.263) still relatively low. We hypothesized that IHD per se would augment the production of BNP in an opposite manner because myocardial ischemia increased the secretion of BNP [23]. Path model B2 examined the cause and effect between the low reactivity of BNP and IHD promotion. Notably, path model B2 revealed an inverse contribution of BNP to IHD (β: -0.556) and a positive contribution of IHD to BNP (β: 0.343). These analyses highlighted the substantial power of the low reactivity of BNP for IHD and showed that the contribution, (β: -0.263) in path model B1 should have been balanced out by its reverse action. The power of the low reactivity of BNP is a substantially strong but concealed risk factor for IHD.

### Possible mechanisms by which obesity induces the low reactivity of BNP

How obesity induces the low reactivity of BNP is not known, but there are several possible mechanisms. The production of BNP is decreased in obese patients with heart failure, which was documented in our previous report involving blood sampling from the coronary sinus and aortic root during cardiac catheterization [25]. The molecular mechanisms are still not clear, but some studies have demonstrated roles of peroxisome proliferator-activated receptor (PPAR)-α and HIF-1α in BNP production. PPAR-α generally suppresses the production of BNP, and it may be activated in obese patients [26, 27]. HIF-1α generally augments the production of BNP, and it is decreased in diabetic patients with obesity [28, 29]. We discussed the precise mechanisms in a previous report [30].

### Possible mechanisms for the promotion of IHD induced by the low reactivity of BNP

These results suggest that the low reactivity of BNP plays a crucial causative role in IHD. The contribution of low-reactive BNP to the advancement of IHD should be examined closely in future studies. The low response of cyclic guanosine monophosphate (GMP) due to low-reactive BNP may be important because cyclic GMP is the second messenger of natriuretic peptides and it contributes to arterial vasodilation [31]. Natriuretic peptides may reduce some adhesion molecules that produce atherosclerosis [32]. The low reactivity of BNP would cause vasoconstriction of the arteries and adherence of leukocytes and other cells, including cancer cells [33].

### Possible relationship with obesity paradox

This study demonstrated that obesity per se is not a strong risk factor for IHD in Japanese patients. This finding may be related partly to the obesity paradox, a recent hypothesis that obesity may be protective and associated with greater survival in certain groups of people, such as very elderly individuals or those with certain chronic diseases, including heart failure [8–10,34]. At a minimum, this study suggests that mild obesity itself is not particularly harmful in the absence of other risk factors.

### Explanation of the BNP assay system

The BNP assay system we used in this study was a rapid enzyme-linked immunosorbent assay (non-extracted) kit using an antibody to human BNP (Shionogi Co. Ltd., Tokyo, Japan), as mentioned in the methods section. In some detail, this BNP assay system detects not only the 32-amino-acid authentic BNP (BNP32), the biologically active form, but also the 108-amino-acid proBNP (1–108), a less active form [35]. To our knowledge, there is no method for the measurement of BNP32 alone. Therefore, a more detailed discussion of the meaning of a ‘high’ plasma BNP level measured with this assay system is necessary. On another front, the current study, demonstrated that ‘low’ plasma levels of BNP were significant. This means that the combined levels of BNP32 and proBNP in the circulation were low, indicating that the amount of BNP32 is naturally small. Therefore, we can deny responsibility for the discussion about molecular forms of BNP in this study. Nonetheless, to confirm the current result, the most accurate method of assessing the biological activity of cardiac endocrine function (including both A-type natriuretic peptide and BNP) would be the simultaneous measurement of cyclic GMP in urine samples [36].

### Possible contribution of gender differences to the current results

In the statistical analysis, other explanatory variables should be taken into consideration, especially,gender differences. It has been reported that plasma BNP levels differ between males and females [24,37]. However, gender differences had a limited influence in this study. Again, we should note that the study population was only mildly obese.

### Abundant remaining risks for IHD estimated by covariance structure analysis

The current statistics using covariance structure analyses were performed for explanatory factor analysis. The predictors of IHD were estimated to explain 18.4% of its variance. In other words, the error variance of IHD is approximately 81.6% of the variance of IHD itself. This result indicates that other factors are associated with the progression of IHD, which require clarification in the future.

### Warning about future obesity in Japan

We should not underestimate the effect of obesity itself on increasing the risk of future episodes, either directly or indirectly because childhood obesity is associated with increased cardiovascular risk both globally [38] and in Japan [39].

### Study limitations

First, as shown in Table 1, the pharmacological treatment differed between the IHD and non-IHD groups. These therapies might have exerted some influence on the current results. We should conduct another study to examine the preventive benefit of drugs. Second, it would be interesting to examine the effects of heart failure types, which are classified as heart failure with reduced or preserved ejection fraction (HFrEF or HFpEF), or to stratify the data by symptomatic vs. asymptomatic patients. We could not perform such an analysis in the current work because we had only limited data on precise diastolic function (measured by echocardiography) and more detailed clinical signs.

## Conclusion

This study used covariance structure analysis and demonstrated that obesity per se was not a significant risk for IHD in Japanese patients. However, several important risk factors triggered by obesity played a causative role in IHD. The low reactivity of BNP was induced by obesity and augmented the progression of IHD.
